# Annotations

**Published:** 1901-06-01

**Authors:** 


### Annotations.

### Dishonest Vaccination.

Medical men are seldom backward in posing as members of a noble profession. Indeed from the day the medical student "enters" and hears his first "introductory" the nobility of our work and the loftiness of our ambitions are so perpetually drummed into him that it is hardly to be wondered at if doctors sometimes regard themselves not quite as other men. So much for profession. How about practice? How are we to reconcile all these boasts with such a low standard of ethics as is shown by the prevalence of a system of vaccination which can only be called dishonest. It is a serious matter that men should be allowed to remain on the Register who persist in performing vaccination in such a manner as must, so far as their knowledge on the subject goes, result in its failing to give that protection against smallpox which is the sole excuse for its being done at all. The only knowledge possessed by the general practitioner in regard to the utility of vaccination is just what he has been taught and nothing else. He accepts what his teachers tell him about the utility of vaccination, and that is his sole excuse for doing it and taking his fee for its performance. Why, then, does he not also accept what they tell him as to the manner in which the operation should be done? and why is the "one-mark" man who does the operation in such a way as to ensure its inutility allowed to remain a member of the profession? That the medical profession is subject to discipline of a sort no one can deny. A medical man who oversteps the line even by giving gas for an unregistered dentist, is liable to be taken off the Register by the General Medical Council as by law established. History has shown that men who choose to act contrary to the desires of their colleagues, even in a town at the Antipodes, may be turned out of the British Medical Association, a body fairly representative of professional feeling; while we all know the professional ostracism which would be dealt out to any of our members who showed themselves weak-kneed on the doctrine of "similars" in regard to the drug treatment of disease. Surely it shows a lack of all sense of proportion—and even of humour—that a noble profession, which in some things has a hand so heavy should deal so lightly with sheer dishonesty as to allow these "one-mark vaccinators" to remain within the pale.

### Why not a State Examination?

In a few days the General Medical Council will meet for the transaction of business, and one of the most vital questions which it will probably have to discuss will be its relations to the Royal Colleges of Physicians and of Surgeons. It is impossible not to see that as things stand at present such discredit is thrown upon the whole system of medical education and qualification in this country as to play into the hands of those who ask that all who wish to practise medicine within the borders of these isles shall pass a State examination, after the fashion which prevails in Germany and other foreign countries. So far as the public is concerned the one prime function of the Medical Council is to maintain the Register, so that everyone shall be able to distinguish between a qualified and an unqualified practitioner. For this purpose it is essential that the Council shall exercise such control over medical education that none shall gain access to the Register who have not been properly taught their trade. When then we find the Colleges defying the Council in a matter of this kind, we feel that they are playing with edge tools. But for the pressure of Parliamentary business it would not take much to upset the whole ridiculous system of medical qualification, with its thirty or forty competing diploma shops, and make the right to practise depend upon the passing of a State examination, and if a strong man were to arise who would voice the demand for reform, this would not easily be resisted. The present state of affairs is tolerable only on condition that the numerous qualifying bodies, which now earn a more or less honest living by providing would-be doctors with diplomas, are kept under such strict control that they shall be prevented from driving a "cutting trade." There is, indeed, but little excuse for the continuance of many of them, and if they make themselves a nuisance, and advance pretensions which are opposed to the public weal, they will have to learn that the world can get on very well without them.

### The Colliery Explosion in South Wales.

The recent disastrous colliery explosion in South Wales offers another example of the dangers connected with the accumulation of coal dust in the workings of mines. The extremely free ventilation to which fiery mines have to be subjected in order to clear them of explosive gases has in turn its own dangers. The air becomes heated as it descends, and especially when dry to begin with, as has recently been the case, its capacity for absorbing moisture becomes very considerable. The coal dust in the workings becomes as dry as tinder, and any disturbance, such as may be caused by a slight and otherwise harmless explosion or even by a small fall of coal or of roof, so charges the atmosphere with microscopic particles of carbon that it becomes as truly explosive as a mixture of gas and air; but far more dangerous. The danger of an explosion in a coal mine, one need hardly say, is not to be measured by the amount of physical destruction which it produces. The risk to man lies mostly in the poisonous effects of the products of combustion—the after-damp. That everyone knows, but it is not so generally recognised that the after-damp from a coal-dust explosion is of a far more dangerous nature than that from a gas or fire-damp explosion. When the explosive compound consists of gas and air there is almost certain to be a considerable excess of oxygen in the mixture, so that the combustion of the carbon is fairly complete, and the product is carbonic acid gas—carbon dioxide—a gas which is poisonous enough if concentrated, but not very injurious if freely mixed with air. When, however, the explosive compound is formed by air and coal dust, and when the wave of the explosion sweeps before it such a cloud of dust that the whole air becomes highly charged with carbonaceous matter, instead of oxygen being in excess carbon is the element that prevails. Thus the carbon is only partially consumed, and the product is the lower form of oxidation, the deadly carbonic oxide or carbon monoxide. This is a positively poisonous gas which, even when well diluted, is very destructive to life. Carbon monoxide owes its extremely dangerous properties to the fact that when inspired it enters into direct combination with the hæmoglobin of the blood, with which it forms so staple a compound that the red blood cells become incapable of carrying oxygen to the tissues. So poisonous is it that the inhalation of an atmosphere containing even only 1 to 2 per cent. of carbon monoxide may cause very serious symptoms. Fortunately, death from this cause is a painless euthanasia.

